# Early predominant inflammatory myopathy in anti‐glycyl‐tRNA synthetase (EJ) antibody positive antisynthetase syndrome

**DOI:** 10.1002/ccr3.3773

**Published:** 2021-01-15

**Authors:** Robin Warner, Derrece Reid

**Affiliations:** ^1^ Department of Neurology Hospital for Special Surgery New York NY USA; ^2^ Spine Care Long Island Plainview NY USA

**Keywords:** antisynthetase syndrome, EJ antibody, Glycyl‐tRNA, myopathy

## Abstract

It is important to consider antisynthetase syndrome in the differential diagnosis of patients presenting with weakness, respiratory distress, and a constellation of complaints spanning multiple organ systems, as this will change clinical management.

## Graphical abstract

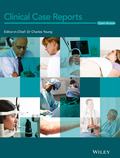

## 1 INTRODUCTION

A 51‐year‐old man presented with weakness and constitutional symptoms and found on laboratories to have anti‐glycyl‐tRNA synthetase (EJ) antibody antisynthetase syndrome. Myopathy is usually a late manifestation of antisynthetase syndrome and is steroid‐responsive; however in our case, it occurred earlier. It is important to consider antisynthetase syndrome in patients presenting with inflammatory myopathy.

Antisynthetase syndrome is a constellation of interstitial lung disease (ILD, 70%), myositis, arthritis (50%), rash (30%), Sicca syndrome, Raynaud's phenomenon, “mechanic's hands” (chapped fingers), and constitutional symptoms.^1^, ^2^, ^3^ Its prevalence is 2:1 000 000.^1^ Antibodies are produced to aminoacyl tRNA synthetases that catalyze attachment of amino acids to tRNA and recruit inflammatory cells to sites of muscle and lung injury.^1^ In EJ antibody antisynthetase syndrome, the antigen is glycyl‐tRNA synthetase (GARS).^2^

Eight anti‐aminoacyl‐tRNA synthetases (ARS) have been described; anti‐EJ comprise 2%‐5%.^3^ Antibody phenotypes are similar, except anti‐PL7 and anti‐PL12 are ILD predominant. Myositis is typically a late manifestation at 17 months and occurs in 59%.^2^, ^4^ A retrospective study of anti‐EJ antisynthetase syndrome revealed 41% of their myositis‐predominant cohort had amyopathic dermatomyositis; the remainder had phenotypes of dermatomyositis or polymyositis.^4^ Long‐term immunosuppression is required 80% of the time.^5^

## 2 CASE REPORT

We present a 51‐year‐old man with 2 years of leg weakness, starting with trouble rising from bed and red papular rash. He was inpatient for 3 weeks with improvement on prednisone. After discharge, he required a wheelchair due to pain and had intermittent aches, fever, and periorbital edema. His past medical history included connective tissue disorder, eczema, costochondritis, arthritis, and sleep apnea.

Examination showed periorbital edema, diminished breath sounds, and papular rash on his extremities. Neurologically, the only finding was 5‐/5 strength of bilateral hip flexors. Electromyography revealed a length dependent, axonal neuropathy, with absent sural SNAPs and low amplitude perineal CMAPs. Quadriceps biopsy showed CD3‐positive cells and atrophic angulated fibers, suggesting neurogenic atrophy. Sural nerve biopsy showed loss of myelinated fibers, regenerative clusters, and thinly myelinated axons. CT chest showed bibasilar ground glass opacities.

Abnormal laboratories were ESR 51, CRP 35.4, CK 1874 (peak 5973), elevated aldolase, and Sjogren's anti‐SSA. A myositis panel revealed positive EJ autoantibodies. The patient was diagnosed with EJ antibody antisynthetase syndrome. He was treated with Cyclosporine, improved significantly, and was lost to follow up.

## 3 DISCUSSION

Antisynthetase syndrome is a multi‐organ syndrome causing myositis, which manifests as symmetric (85%) proximal (60%) weakness, acute pain (34%‐50%), progressing to muscle atrophy and fibrosis in 66%, occurring at average age 56.^3^, ^4^ Weakness of cricopharyngeus and hypopharynx can lead to dysphagia 15%‐40% of the time.^1^ Spirometry can reveal weakness in the diaphragm or the intercostal muscles.^1^

Anti‐aminoacyl‐tRNA synthetase antibodies are detected in 25%‐35% of patients with idiopathic inflammatory myopathy. ARS enzymes may recruit antigen‐presenting cells to sites of muscle and lung injury. However, the enzyme's function has not been linked to the disease process. The reason or mechanism by which ARS antibody production occurs is unknown.^1^ Myopathy is detected by electrodiagnostic or laboratory evidence.^1^, ^6^ Steroid‐responsive proximal myopathy is usually a late manifestation of the disease course (17 months); however, here it occurred on presentation.^1^, ^5^ A recent study revealed dermatomyositis as the most common myopathy of anti‐EJ antisynthetase syndrome.^4^ 41% of those were amyopathic, meaning myopathy was absent or extremely mild.^4^

Laboratories reveal elevated muscle enzymes, positive ANA, antisynthetase antibodies, and elevated acute‐phase reactants.^1^ CKs can be followed to assess myositis activity.^7^ Anti‐Ro52 antibodies, which are associated with myositis, accompanied anti‐EJ antibodies in 92% of patients in one study.^4^ Electromyography shows myopathy, but up to 15% of tests are normal.^1^ Muscle MRI shows edema not limited to compartment or myotome.^8^

Muscle biopsy shows perimysial macrophages and lymphocytes, degenerating muscle fibers and regenerating muscle fibers, muscle fiber necrosis, and perifascicular atrophy similar to dermatomyositis, but without vascular changes.^1^ EJ antibodies produce a cytoplasmic pattern on immunofluorescence.^1^

Corticosteroids produce an incomplete response.^5^ Long‐term immunosuppressant medications, such as Rituximab, Cyclophosphamide, IVIG, or Methotrexate, are required 80% of the time.^5^ 75% of patients respond to Rituximab at 375 mg/M^2^ every 10‐12 weeks.^1^

It is important to consider antisynthetase syndrome in the differential diagnosis of weakness and signs of multi‐organ disease, often diagnosed as multiple diseases. Successfully diagnosing antisynthetase syndrome results in better patient outcomes.

## CONFLICT OF INTEREST

Dr Warner reports no disclosures. Dr Reid reports no disclosures.

## AUTHOR CONTRIBUTION

Dr RW: collected all clinical information and wrote the paper. Dr DR saw and examined the patient, analyzed the clinical information and performed a literature search that resulted in diagnosis. She oversaw writing and editing of this paper.

## ETHICAL APPROVAL

This material is the authors' own original work, which has not been previously published elsewhere. The paper is not currently being considered for publication elsewhere. The paper reflects the authors' own research and analysis in a truthful and complete manner. Written informed consent was obtained from the participant of this study.

## Data Availability

Data sharing not applicable to this article as no datasets were generated or analyzed during the current study.
